# Supplementary material for: FFixR: a machine learning framework for accurate somatic mutation calling from FFPE RNA-seq data in cancer
Source: Bioinformatics. 2026 Jun 24;42(7):btag405. doi: 10.1093/bioinformatics/btag405 (PMC13335478; doi:10.1093/bioinformatics/btag405)
Supplement: btag405_Supplementary_Data [file btag405_supplementary_data.zip › supplementary_figures_Livne_et_al.docx]

Supplementary figures

**
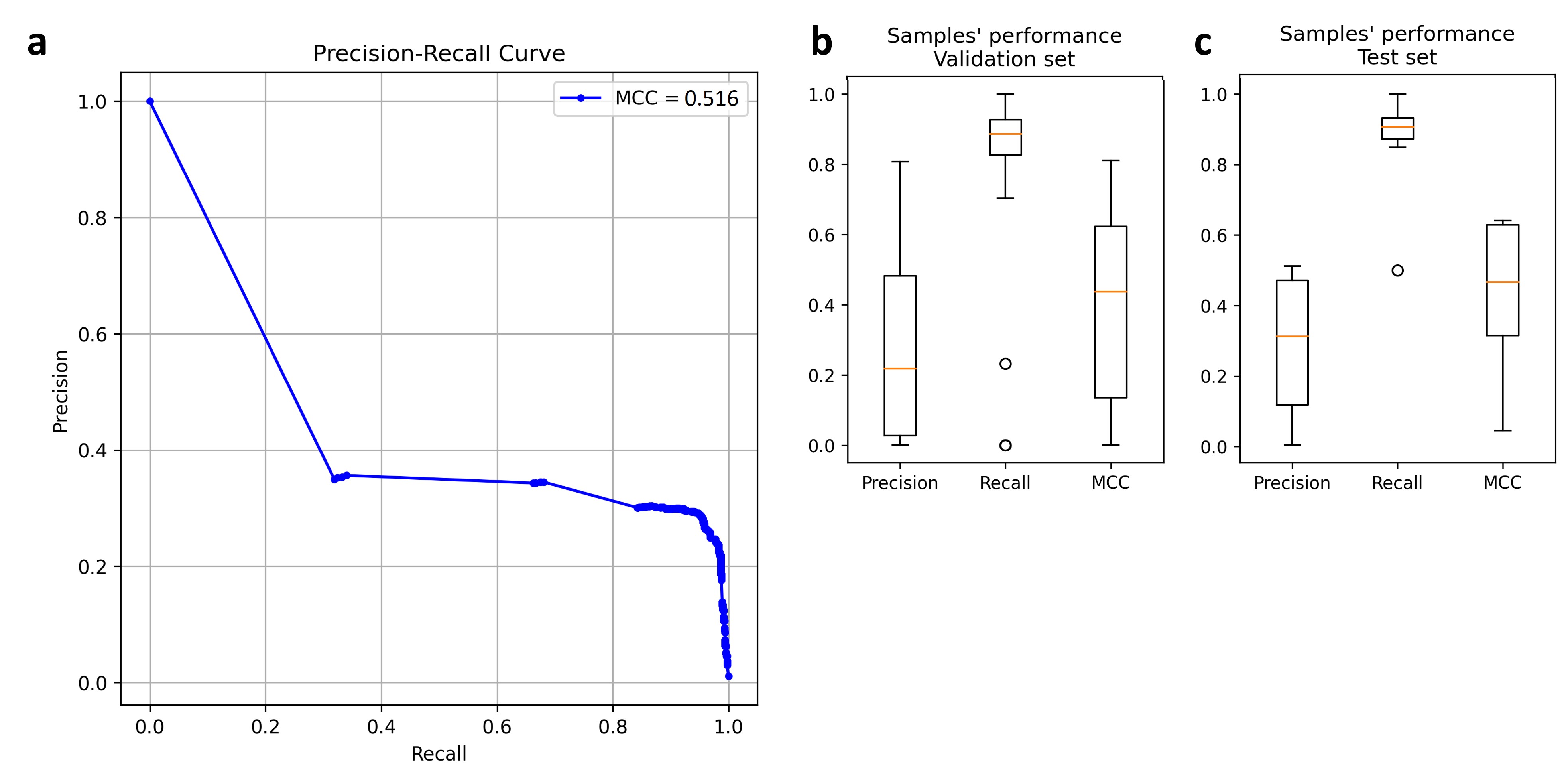
**

**Supp. Fig 1|** **Model’s performance excluding the features signatures 1, 30.** **a** Precision-Recall curve based on the model excluding the features signatures 1, 30. **b** Distribution of precision, recall and MCC values on the validation (b) and test (c) sets. Box plots show median, 25th, and 75th percentiles. The whiskers extend to the most extreme data points not considered outliers, and the outliers are represented as dots.

**
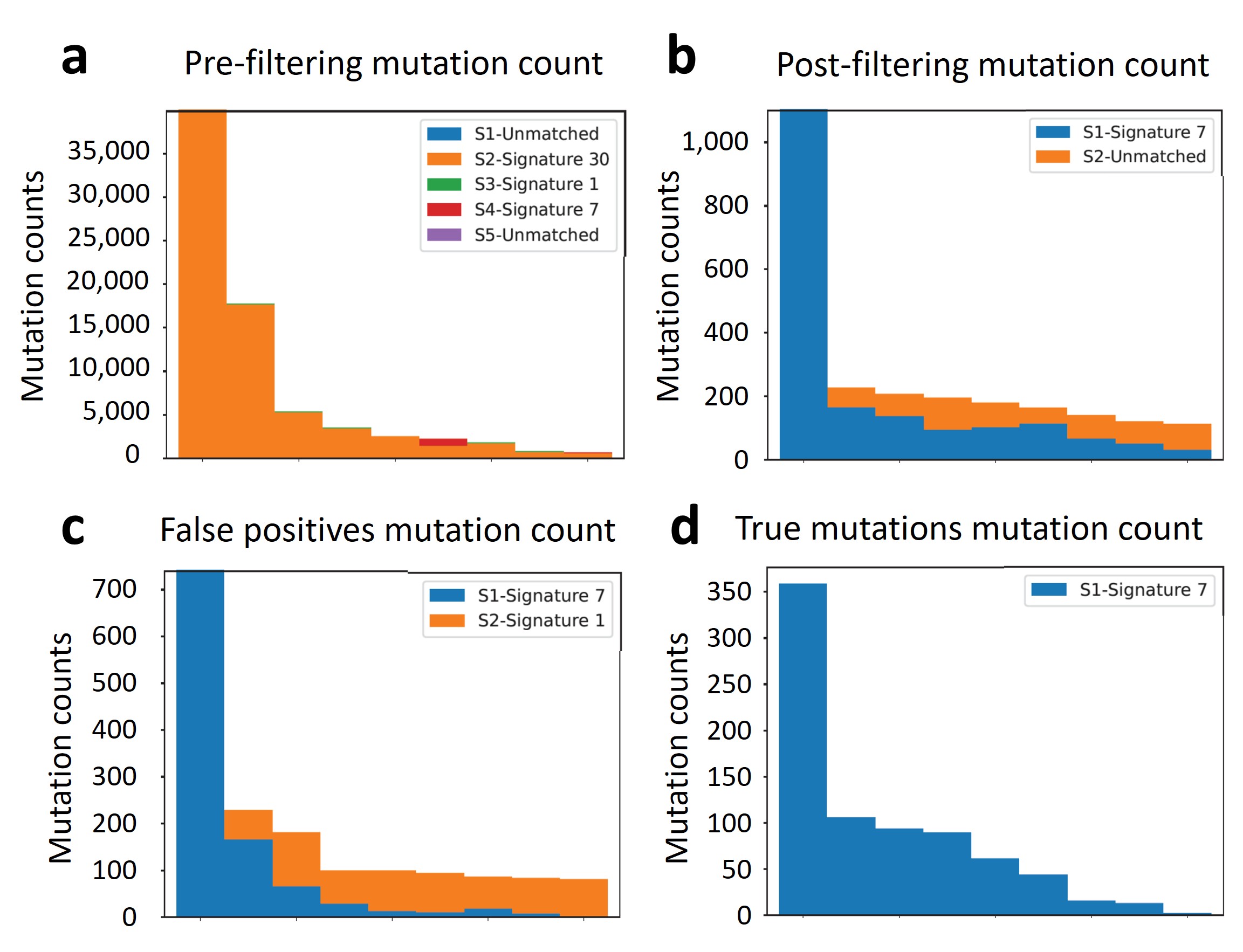
**

**Supp. Fig. 2| Signature analysis for the model trained without signatures 1 and 30 features. a-e.** Mutation count and their association with mutational signatures for pre-filtering mutations (a); post-filtering mutations (b); false positive mutations (c); true mutations (d).

**
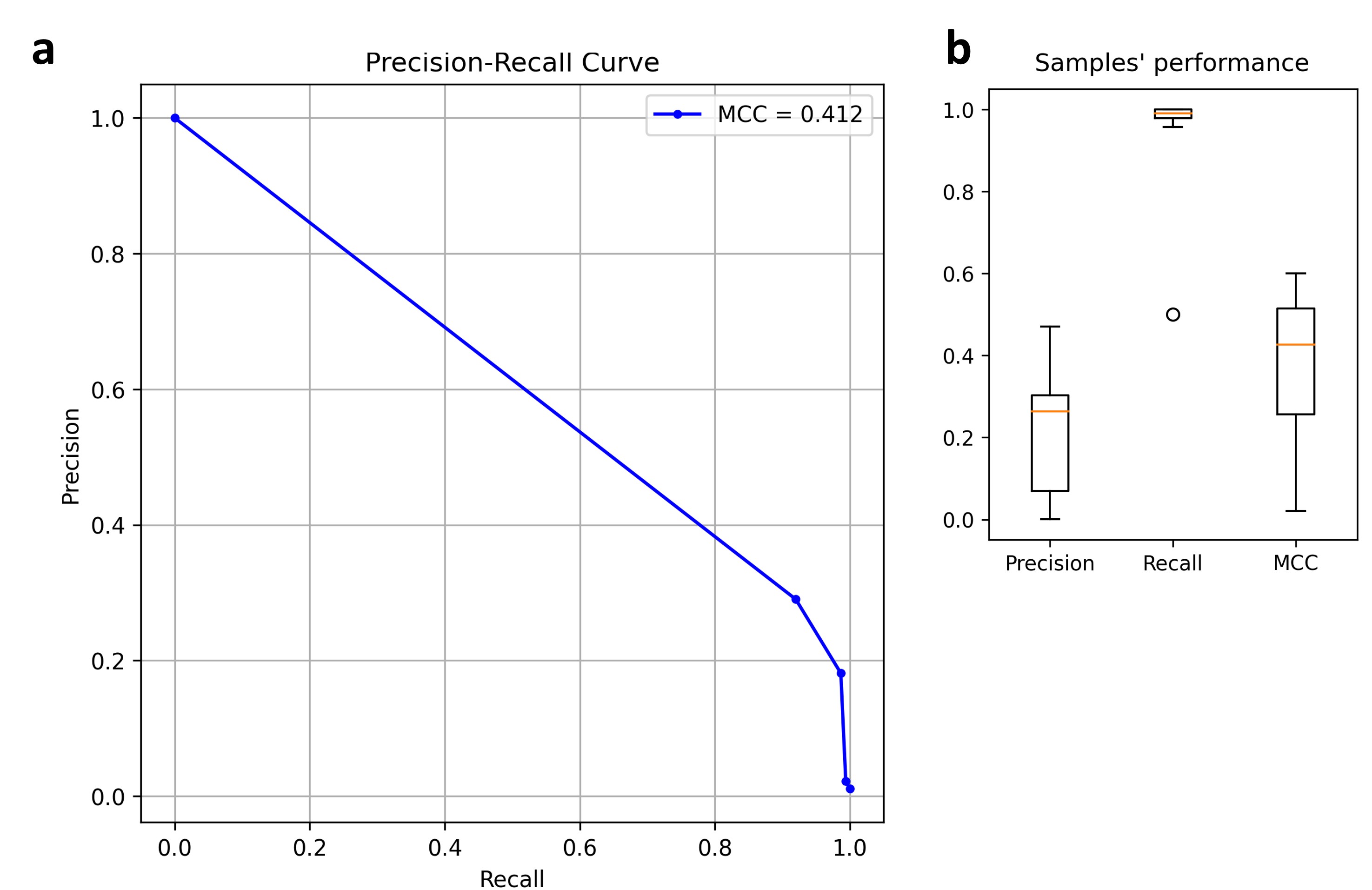
Supp. Fig. 3| Performance based on the 3 most significant features thresholds alone.** **a** Precision-Recall curve based on the 3 most significant features thresholds alone. **b** Distribution of precision, recall and MCC values. Box plots show median, 25th, and 75th percentiles. The whiskers extend to the most extreme data points not considered outliers, and the outliers are represented as dots.

**
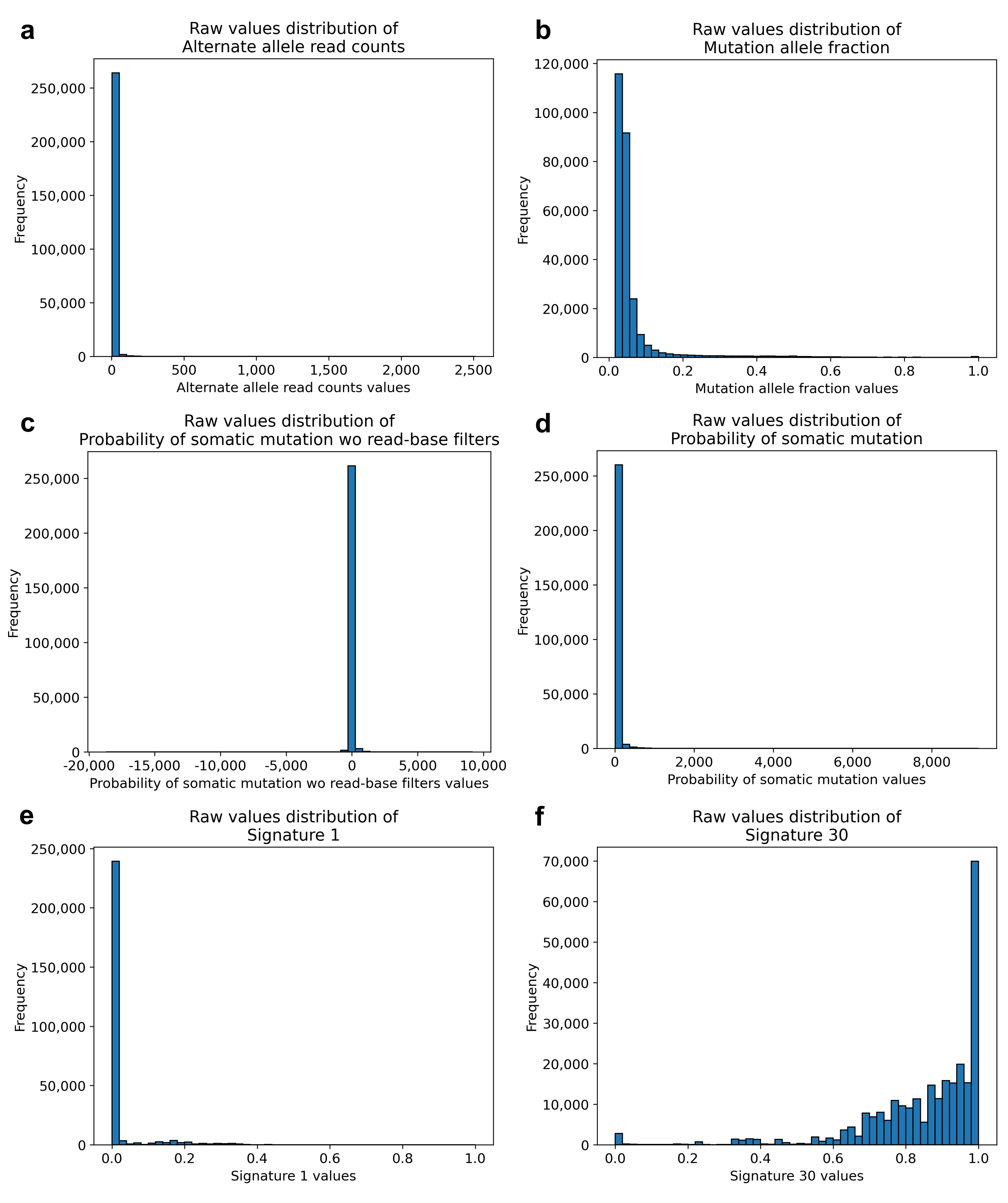
Supp. Fig. 4| Numerical features raw values.** **a** Raw values distribution of alternate allele read count. **b** Raw values distribution of mutation allele fraction. **c** Raw values distribution of probability of somatic mutation without read-base filters. **d** Raw values distribution of probability of somatic mutation. **e** Raw values distribution of signature 1. **f** Raw values distribution of signature 30.

**
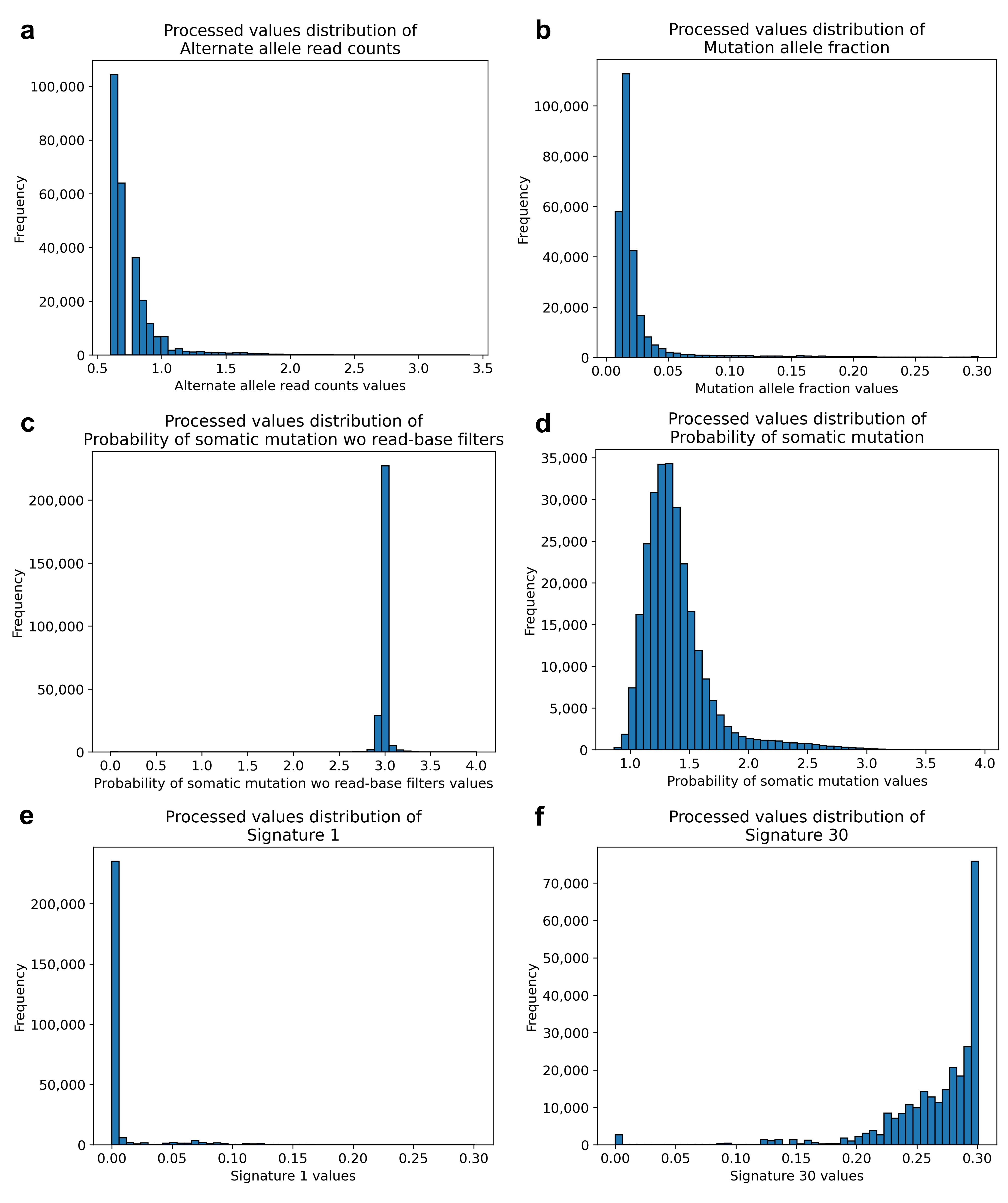
Supp. Fig. 5| Numerical features processed values.** **a** Processed values distribution of alternate allele read count. **b** Processed values distribution of mutation allele fraction. **c** Processed values distribution of probability of somatic mutation without read-base filters. **d** Processed values distribution of probability of somatic mutation. **e** Processed values distribution of signature 1. **f** Processed values distribution of signature 30.

**
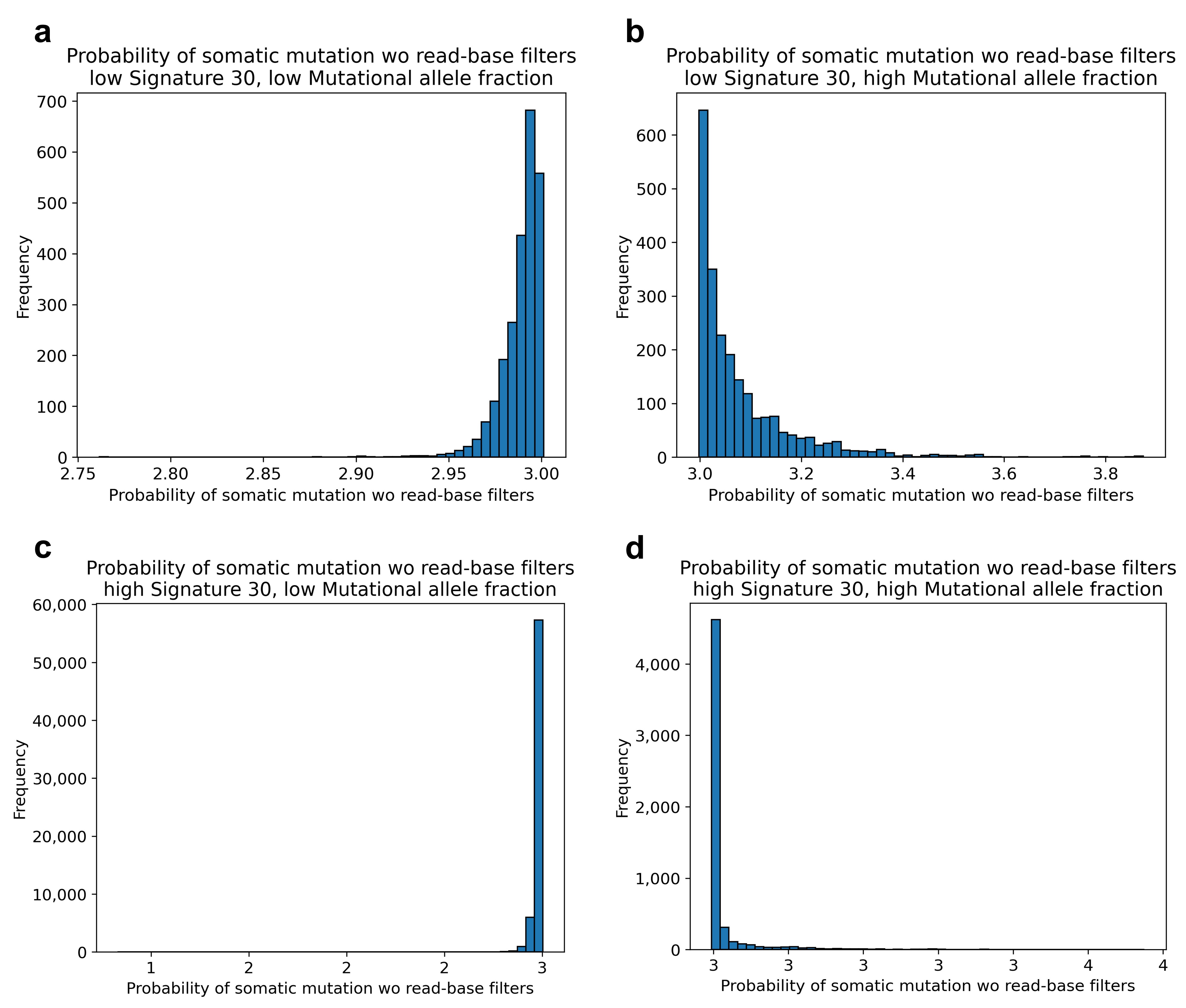
**

**Supp. Fig. 6| Values of Probability of somatic mutation without read-base filters for the various combinations of High/ Low values of Signature 30 and Mutation allele fraction features.** **a** Probability of somatic mutation without read-base filters for low signature 30 and low mutational allele fraction. **b** Probability of somatic mutation without read-base filters for low signature 30 and high mutational allele fraction. **c** Probability of somatic mutation without read-base filters for high signature 30 and low mutational allele fraction. **d** Probability of somatic mutation without read-base filters for high signature 30 and high mutational allele fraction.

**
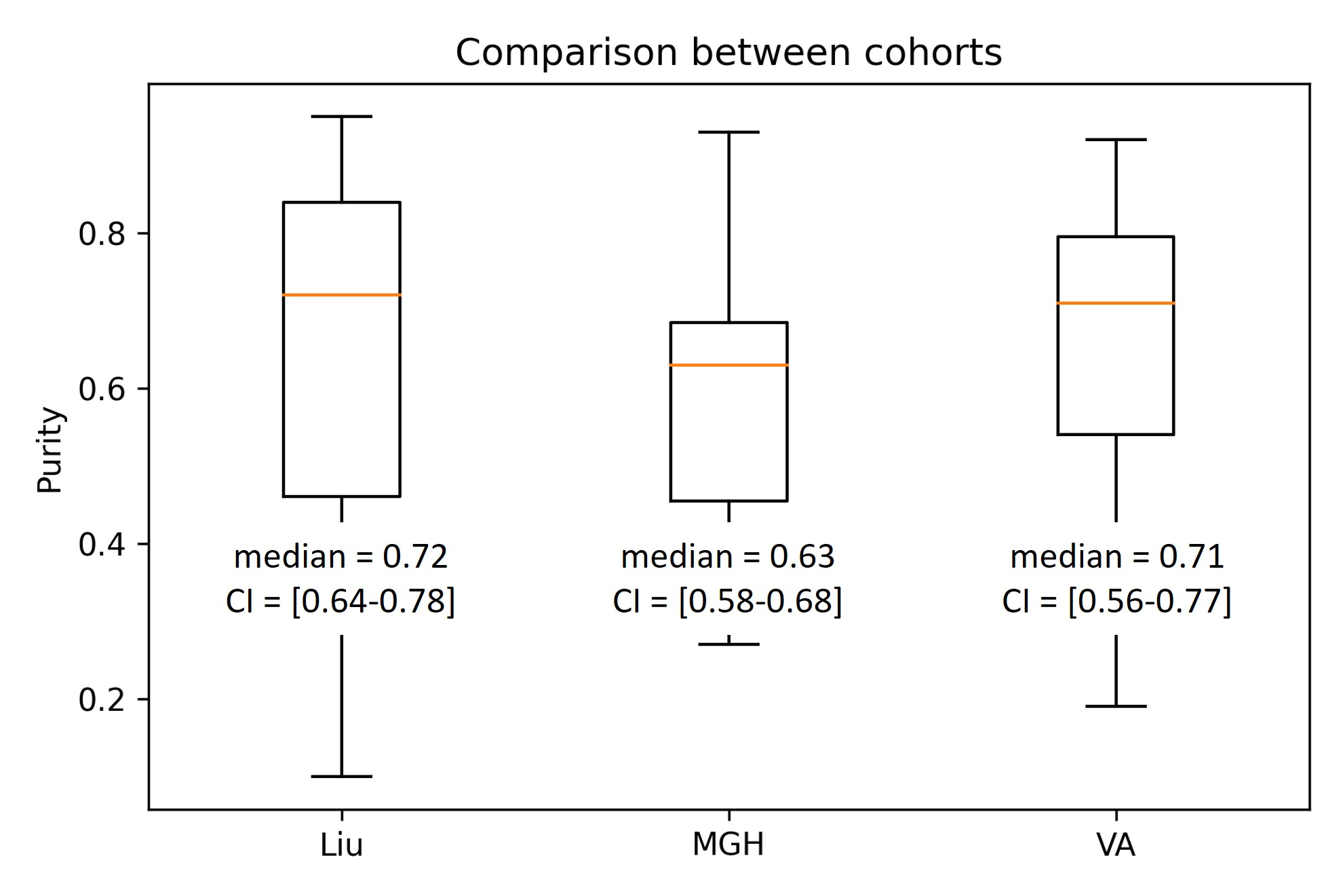
Supp. Fig. 7| Comparing tumor purity values between the various cohorts.** Distribution of tumor purity values in the Liu et al, MGH (Freeman et al.) and Val Allen et al. cohorts.

**
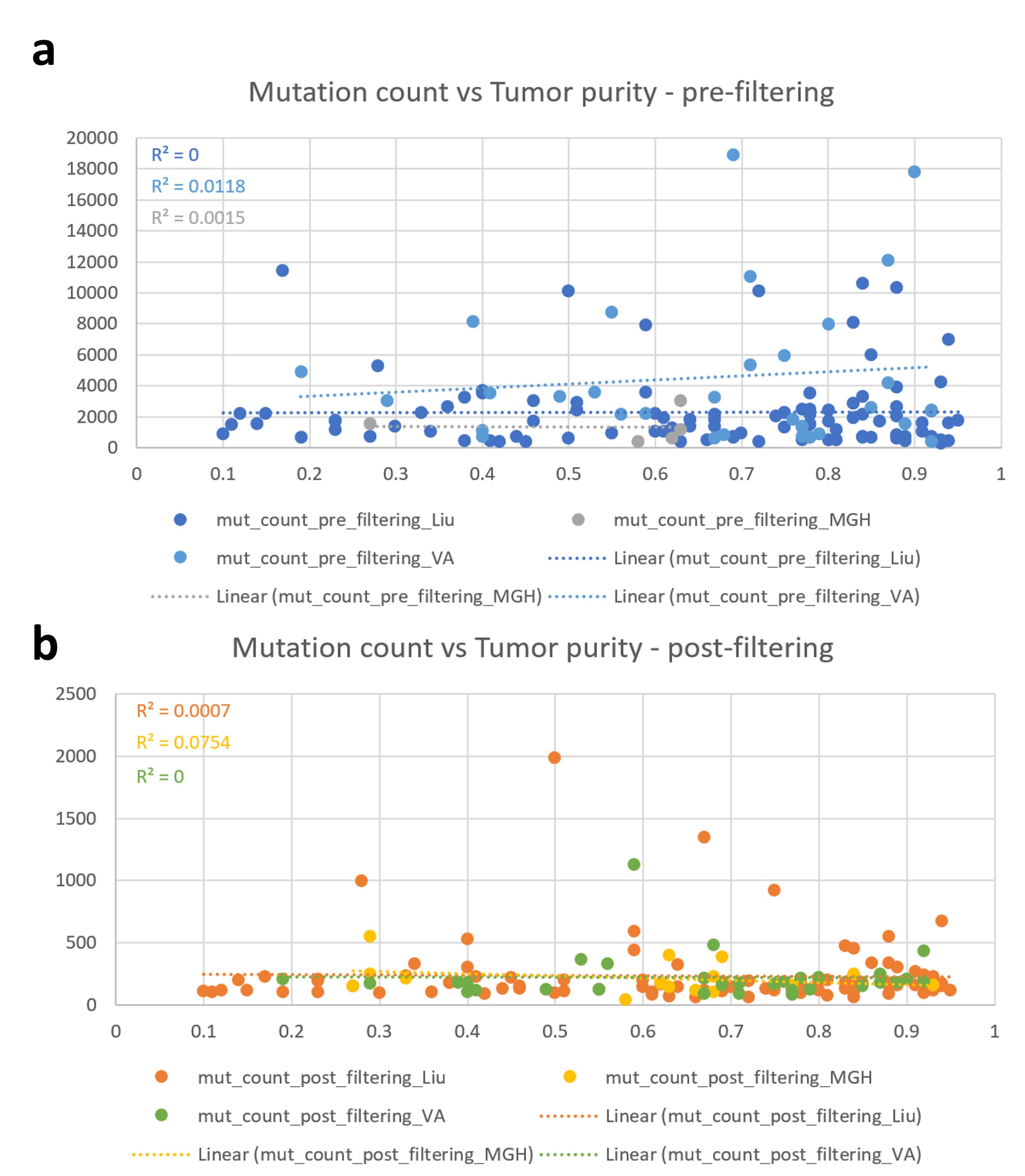
 Supp. Fig. 8| Mutation count versus tumor purity over the various cohorts.** **a** Presenting the mutation counts and tumor purity for each patient of the various cohorts, pre-filtering. The equations show the mathematical correlation between the values, and the R^2^ correlation. **b** Presenting the mutation counts and tumor purity for each patient of the various cohorts, post-filtering. The equations show the mathematical correlation between the values, and the R^2^ correlation.
